# Coupling of Cell Division and Differentiation in *Arabidopsis thaliana* Cultured Cells with Interaction of Ethylene and ABA Signaling Pathways

**DOI:** 10.3390/life10020015

**Published:** 2020-02-10

**Authors:** Galina V. Novikova, Natalia S. Stepanchenko, Anna A. Zorina, Alexander V. Nosov, Victor Y. Rakitin, Igor E. Moshkov, Dmitry A. Los

**Affiliations:** K.A. Timiryazev Institute of Plant Physiology, Russian Academy of Sciences, Botanicheskaya Street 35, Moscow 127276, Russian

**Keywords:** *Arabidopsis thaliana*, abscisic acid, cell culture, cell differentiation, cell proliferation, ethylene

## Abstract

Recent studies indicate direct links between molecular cell cycle and cell differentiation machineries. Ethylene and abscisic acid (ABA) are known to affect cell division and differentiation, but the mechanisms of such effects are poorly understood. As ethylene and ABA signaling routes may interact, we examined their involvement in cell division and differentiation in cell tissue cultures derived from several *Arabidopsis* *thaliana* plants: wild type (Col-0), and ethylene-insensitive mutants *etr1-1, ctr1-1,* and *ein2-1*. We designed an experimental setup to analyze the growth-related parameters and molecular mechanisms in proliferating cells upon short exposure to ABA. Here, we provide evidence for the ethylene–ABA signaling pathways’ interaction in the regulation of cell division and differentiation as follows: (1) when the ethylene signal transduction pathway is functionally active (Col-0), the cells actively proliferate, and exogenous ABA performs its function as an inhibitor of DNA synthesis and division; (2) if the ethylene signal is not perceived (*etr1-1*), then, in addition to cell differentiation (tracheary elements formation), cell death can occur. The addition of exogenous ABA can rescue the cells via increasing proliferation; (3) if the ethylene signal is perceived, but not transduced (*ein2-1*), then cell differentiation takes place—the latter is enhanced by exogenous ABA while cell proliferation is reduced; (4) when the signal transduction pathway is constitutively active, the cells begin to exit the cell cycle and proceed to endo-reduplication (*ctr1-1*). In this case, the addition of exogenous ABA promotes reactivation of cell division.

## 1. Introduction

The structure and function of major signaling hubs for intracellular plant growth regulators is one of the longstanding biological questions. In plants, cells divide when triggered by extracellular or intracellular stimuli, including phytohormones. Proliferative signals flow through intracellular signaling pathways to activate the cell cycle. Therefore, cells rely on proliferative signaling pathways to regulate entry into the cell cycle.

Plant growth, which depends on cell division as one of the critical processes, is regulated by several hormones. Taking this into account, one can assume that regulation of the cell cycle is a result of cross-talk of hormone signal transduction pathways. It is generally accepted that phytohormones are common regulators of cell cycle, with auxins and cytokinins being the most extensively documented [1], while the significance of ethylene and abscisic acid (ABA) in this process is still a matter of debate, and there are no data on the cross-talk of signaling pathways of these hormones in regards to cell proliferation.

It is known that, in stressed intact plants, ethylene and ABA influence each other’s synthesis [2], and their signal transduction paths can cross-talk [3,4,5]. However, there is no information on interaction of the signaling pathways for ethylene and ABA in non-stressed plants.

Previously, we demonstrated that cell tissue culture derived from *Arabidopsis thaliana*, both wild type and ethylene insensitive mutants, is an appropriate model to study ethylene and ABA effects on cell division under non-stressed conditions [6].

It is fair to say that more is known about ethylene signal perception and transduction than signal perception and transduction for any another plant hormone. The data on ethylene signaling are generated mostly from experimental work on ethylene sensitivity mutants. Using the mutants and identifying the genes where the lesions are, the ethylene transduction chain has been revealed. These involve five partially functionally redundant receptors, localized in the endoplasmic reticulum (ER) and Golgi membrane: ERS1 (ETHYLENE RESPONSE SENSOR 1), ERS2, ETR1 (ETHYLENE RESISTANCE 1), ETR2 and EIN4 (ETHYLENE INSENSITIVE 4). Downstream from the ethylene receptors, there is CTR1 (Constitutive Triple Response 1), a protein homologous to MAPKKK of Raf-type. One particular feature of the ethylene signaling pathway is that the receptors and CTR1 are negative regulators. In a free form, the receptors are bound to CTR1 and this module is permanently in an ‘ON’ state, suppressing ER-localized downstream protein EIN2, which may be an ion transporter, and transcription activators from EIN3 series. Upon ethylene binding, the receptors and CTR1 are switched ‘OFF’, which releases the rest of the pathway from inhibition, thus initiating ethylene responses [7].

Genetic screens have identified many mutants with an altered response to exogenous ABA. These approaches have led to a complex and unexplainable pattern of signal transduction. The hunt for ABA receptors succeeded with a new family of proteins called REGULATORY COMPONENTS OF ABA RECEPTOR (RCAR)/PYRABACTIN RESISTANCE1 (PYR1)/PYR1-LIKE (PYL), and a number (clade A) of protein phosphatases of type 2C (PP2C), which function as coreceptors [8,9]. When ABA binds to the receptors, the active site of PP2C is blocked and three associated sucrose non-fermenting-1 (SNF1)-related protein kinase 2s (SnRK2s) are released from PP2C inhibition [10,11,12,13]. Then, SnRK2s auto-activates and can subsequently phosphorylate and activate downstream transcription factors, such as ABA-RESPONSIVE ELEMENT (ABREs)/ABRE-BINDING FACTORS (ABFs), to initiate transcription of the stress-responsive and/or ABA-responsive genes controlled by the SnRK2s [14,15]. In multicellular organisms, cell division is strictly regulated to avoid uncontrolled cell proliferation, as well as to allow cells to escape from the cell cycle in order to differentiate in accordance with their developmental program. The eukaryotic cell cycle is composed of four well-defined phases, G1, S, G2 and mitosis (M). Progression through the cell cycle is strictly regulated, especially during transitions from the G1 phase to S phase, from G2 phase to M and when leaving M to return to G1. Most eukaryotic cells divide only in the presence of mitogens, triggering cells to pass through this control point of the G1/S cell cycle. Although the core cell cycle machinery is rather conserved in both animals and plants [16], the nature of the mitogens, as well as the associated signaling pathways, differ substantially.

There is evidence that ethylene and ABA can act as mitogens. It was shown that ethylene inhibited the replication of nuclear DNA and cell division in pea seedlings [17] and induced programmed cell death at particular phases of the cell cycle in tobacco TBY-2 cell line [18]. In addition, ethylene caused endoreduplication [19], and also stimulated the division of stem cells of quiescent center in root meristem of *Arabidopsis* [20] and cambial cells of poplar [21], together with the cells of quiescent center in maize roots after the excision of the root tip [22]. During osmotic stress, a cell cycle arrest was observed in *A. thaliana* leaves in parallel with an increase in 1-aminocyclopropane-1-carboxylate (ACC) levels and activation of ethylene signaling [23].

Treatment of cultured BY2 cells with ABA arrested the cells between G1 and S, but did not affect other phases of the cell cycle [24]. It should be emphasized that ABA treatment of alfalfa leaves reduced the positive effect of auxin and cytokinins on cell division [25]. ABA generated during abiotic stresses in the roots of *Arabidopsis* decreased the number of divisions [26]. In ABA-deficient mutants of *Arabidopsis*, *aba2/gin1*, the growth of cotyledons, rosettes, stems, roots, and pods was markedly retarded under non-stressful conditions, while ABA treatment stimulated the growth [27]. Similar results were obtained for ABA-deficient mutants of tomato [28].

The *Arabidopsis* leaves at a very early stage, in which cells are proliferating only, are extremely small. Thus, it is technically challenging to analyze the molecular basis of ethylene/ABA-induced cell division with a sufficient developmental and temporal resolution. We designed an experimental setup to enable a simultaneous analysis of growth-related parameters and molecular mechanisms specifically in the proliferating cells upon short-time exposure to ABA. In our study, *Arabidopsis* cell suspension cultures have been used as a model to avoid the problems associated with the analyses of intact plants. Here, we show that cell cultures are suitable for identifying the differential roles of ABA and ethylene in cell proliferation and differentiation. We also demonstrate that the functional activity of proteins that perceive and transduce the ethylene signal determines the relationship between proliferation, differentiation, and cell death, and the latter can be reversed by ABA.

## 2. Materials and Methods

*Plant material and cell* culture treatments. Four genotypes of *Arabidopsis thaliana* (L.) Heynh of heterotrophic suspension cultures, namely, wild type (ecotype Columbia, Col-0), *constitutive triple response1-1 (ctr1-1)* mutant, *ethylene resistant1-1(etr1-1)* and *ethylene insensitive2-1 (ein2-1)* mutants were used. Suspension cell cultures were generated from the plants by A.V. Nosov and deposited into the All-Russia Collection of Cultivated Cells of Higher Plants (http://www.ippras.ru/cfc/alccmp/).

Cell lines were cultured in the dark in 50-mL Schenk and Hildebrandt medium [29] supplemented with 3% sucrose, 1 mg/L 2,4-Dichlorophenoxyacetic acid (2,4-D; Sigma, St. Louis, MO, USA) and 0.1 mg/L kinetin (Sigma). The cell cultures were agitated on a rotary shaker at 110 rpm at 26 °C and 70% humidity in the dark. At 10 day intervals, a 5 mL aliquot of each culture was transferred to a fresh medium. The growth of suspension cultures was determined by a gravimetric method.

On the 4th day of subculture, cells were treated for 3 hours with a final concentration of either 25 μM ABA or 150 μM 5-bromo-2’-deoxyuridine (Bromodeoxyuridine, BrdU), which is a synthetic analog of thymidine. When a mutual effect of ABA and BrdU was studied, the cell cultures were treated first with ABA for one hour, then BrdU was added, and cultures were incubated with both substances for another two hours. After that, the cells were collected by centrifugation for 5 min at 1400 × *g*, frozen in liquid nitrogen and stored at −70 °C.

*Microscopic analysis of living cells and tracheary elements (TE)*. Cells viability was estimated by the number of cells unstained with 0.02% aqueous solution of Erythrosin B (Sigma-Aldrich, USA). Both living cells and tracheary elements (TEs) were counted using a Fuchs–Rosenthal chamber. Observation of a secondary wall thickening under a light microscope allows TEs to be easily detected [30].

*The incorporation of BrdU into genomic DNA* isolated from cultured cells using the GenElute Plant Genomic DNA Miniprep Kit (Sigma) was quantified as previously described [31]. Genomic DNA (2 μg) was denatured with 0.4 N NaOH and then neutralized by 1 M Tris-HCl (pH 6.8). A neutralized single-stranded DNA (50 ng) solution was applied to a Hybond-C Extra nitrocellulose membrane (45 μm) using a Bio-Dot SF Microfiltration apparatus (Bio-Rad, USA). The membrane was air dried and the DNA was fixed with UV light. The membrane was incubated with mouse monoclonal antibodies against BrdU (Sigma). For visualization, anti-mouse antibodies conjugated with horseradish peroxidase (Promega, USA) were used.

*Ethylene emission* was determined using a Color 106 Gas Chromatograph (Russia) equipped with a flame ionization detector and a device for concentration of hydrocarbons [32,33].

*Determination of ABA content in cultured cells*. In order to determine ABA content 0.5–1.5 g FW of cultured cells were fixed in liquid nitrogen and homogenized in 10 volumes of methanol (–20 °C) containing 10 mg/L 2,6-Di-tert-butyl-4-methylphenol (Sigma-Aldrich) as an antioxidant. The extract was filtered through a GF/F glass filter (Whatman). The GF/F filter was washed (three times) with 90% methanol, all filtrates were combined, and vacuum evaporated.

ABA was purified by anion exchange chromatography on DEAE-Toyoperl 650 M (9 × 40 mm, Toyo Soda MGH) equilibrated with 0.25 M potassium acetate after that with 0.2 N acetic acid, and with 90% methanol. After filtrate loading, the column was washed with 90% methanol and finally with deionized water. Then, an anion exchange column was connected to Chromosorb LC-7 (C18) (6 × 30 mm, Sigma). ABA was eluted with 0.3 N acetic acid. Thereafter, this column was washed with 0.004 N formic acid (pH 3) and connected with Sephasorb HP Ultrafine (4.6 × 250 mm, Pharmacia). ABA was eluted with 0.003 N formic acid in 98% methanol. The ABA retention time was 10 min. The ABA was collected and dried in a nitrogen stream at 40 °C.

The purified dried ABA was methylated by the addition of 200 µl of a diazomethane produced from nitrosomethylurea in a diazomethane generator (Wheaton Scientific). The methylation was carried out at 0 °C for 10 min, the sample was dried at 35 °C in a nitrogen stream, dissolved in 50 μL of hexane, and 1–2 µL was analyzed by gas chromatography.

For the quantification analysis of ABA methyl ester, a gas chromatograph Gasochrome 1109 (Russia) with an electron capture detector with a ^63^Ni radiation source in an HP–17 column (0.53 mm × 10 m, Hewlett-Packard) was used. The injector temperature was 275 °C, the detector—260 °C, and the column—250 °C. The ABA methyl ester retention time was 6 min.

For ABA recovery, the internal standard was utilized. As an internal standard 1 pg per 1 g FW of cultured cells [^3^H]ABA (specific activity 2.55 TBq/mol, Amersham, UK) was applied. Using this purification technique, a 93%–yield of ABA was achieved.

*Isolation of soluble proteins*. Cells frozen in liquid nitrogen were homogenized and proteins (1:1.5, w/vol) were extracted into 50 mM Tris-HCl (pH 7.6), which contained 10 mM MgCl_2_, 2 mM EDTA, 1 mM DTT, 1 mM phenylmethylsulfonyl fluoride (PMSF), 1 mM EGTA, 2 mM Na_3_VO_4_, 1 mM benzamidine, 10 mM NaF, 50 mM β-glycerophosphate and 250 mM sucrose. Soluble proteins obtained by centrifugation (130,000 × *g* for 3 h) were desalted by elution with 10 mM Tris-HCl (pH 7.6) of NAP-5 columns (GE Healthcare Life Science). Protein content was determined with BCA Protein Assay Kit (Sigma).

*Phosphorylation of cytosolic proteins in vitro and visualization of phosphorylated proteins*. Phosphorylation reaction was performed in a reaction mixture containing 20 mM Tris-HCl (pH 7.6), 10 mM MgCl_2_, 1 mM MnCl_2_, 1 mM EGTA, 1 mM DTT, 1 mM PMSF, 2 mM Na_3_VO_4_, 10 mM β -glycerophosphate, 1 mM benzamidine, 10 μM ATP and 18.5 kBq [γ-^32^P]ATP (specific activity 110 TBq/mmol) per 1 μg protein. The reaction was initiated by 50–150 μg of protein and was carried out for 20 min at 30 °C.

Proteins were precipitated at –20 °C with 80% acetone, purified using a DE CleanUp Kit (Bio-Rad), and dissolved in a buffer containing 7.5 M urea, 2 M thiourea, 1% Triton X-100, 4% CHAPS, 20 mM DTT, and 0.2% BioLyte 3/10 Ampholyte (Bio-Rad). In the first dimension, proteins were resolved using a 7 cm Immobiline DryStrip gels (IPG strips) pH 4–7, as recommended by GE Healthcare. In the second dimension, denaturing gel electrophoresis was performed [34]. Gels were stained with silver nitrate according [35], dried, and exposed with a Biomax MR film (Kodak, CA, USA).

*The in vitro determination of MAP kinase activity* was carried out by incubating 10 μg proteins for 20 min at 30 °C in a reaction mixture containing 0.25 mg/mL Myelin Basic Protein (MBP) as an exogenous substrate, 20 mM Tris-HCl (pH 7.6), 10 mM MgCl_2_, 1 mM MnCl_2_, 1 mM EGTA, 1 mM DTT, 1 mM PMSF, 2 mM Na_3_VO_4_, 10 mM β-glycerophosphate, 1 mM benzamidine, 10 μM ATP and 37 kBq of [γ-^32^P]ATP (specific activity 110 TBq/mmol). The reaction was terminated with SDS–PAGE sample buffer. Then SDS–PAGE was carried in a 15% gel. To visualize phosphorylated MBP, dried stained gels were exposed to a Biomax MR X-ray film (Kodak).

*In situ MAP kinase activity* was determined in 10% gel with 0.5 mg/mL MBP polymerized within the gel. After electrophoresis, the gels were incubated in 20% isopropanol, 50 mM Tris-HCl (pH 8.0) and 5 mM 2-mercaptoethanol, followed by washing in 50 mM Tris-HCl (pH 8.0) with 5 mM 2-mercaptoethanol. Then the proteins were re-denatured with 6 M guanidine hydrochloride in 50 mM Tris-HCl (pH 8.0) with 5 mM 2-mercaptoethanol. Protein renaturation was performed in 50 mM Tris-HCl (pH 8.0), 0.04% Tween 40, and 5 mM 2-mercaptoethanol. Then, the gels were preincubated in 40 mM Hepes-NaOH (pH 8.0) containing 2 mM DTT, 10 mM MgCl_2_, 0.1 mM EGTA, and 1 mM MnCl_2_. For phosphorylation reaction, the gels were incubated in 40 mM Hepes-NaOH (pH 8.0) containing 40 μM ATP, 10 mM MgCl_2_, 1 mM MnCl_2_ and 74 kBq/mL [γ-^32^P]ATP (specific activity 110 TBq/mmol). The reaction was terminated with 5% TCA with 1% Na_4_P_2_O_7_. The dried gels were exposed with a Biomax MR X-ray film (Kodak).

*Fractionation of soluble protein mixtures by Liquid-Phase Isoelectric Focusing*. BioLyte 3/10 (Bio-Rad) were mixed with 2.5 mL soluble protein fraction (1.5 mg protein) to a final concentration of 1.5%. The resulting mixture was loaded in an isoelectric focusing column, which was placed between two membranes moistened, respectively, with catholyte (0.1M H_3_PO_4_) and analyte (0.1M NaOH). Focusing was carried out in a MicroRotofor Liquid-Phase IEF Cell (Bio-Rad) according to the manufacturer’s protocol at 10 °C, a power of 1 W and a maximum voltage of 1000 V until the voltage stabilization. Protein fractions (200 μL) were collected using a special device equipped with a vacuum pump. In each of the 10 fractions obtained, the pH value as well as the protein content were measured [36].

*Sample preparation and mass spectrometry*. Samples were prepared for mass spectrometry (MS) using in-gel digestion. After the 2D electrophoresis and/or fractionation of soluble protein by Liquid-Phase Isoelectric Focusing proteins were stained with colloidal CBB G-250 [37]. Gel spots/lanes were excised, cut into equally sized bands and each band was further cut into 1 mm cubes. The gel pieces were destained with 50 mM ammonium bicarbonate in 50% (v/v) acetonitrile. Samples were reduced with 10 mM dithiothreitol at 56 °C for 1 hour and subsequently alkylated with 5 mM iodoacetamide for 45 min at room temperature in the dark. Gel bits were dehydrated in acetonitrile and dried. Sequencing grade modified trypsin (Promega) in 50 mM ammonium bicarbonate, was added to each gel band (1:50 (w/w) protease protein) and digestion was allowed to proceed at 37 °C for 16 h. The tryptic digests were analyzed by MS. MS spectra were obtained on a tandem MALDI time-of-flight mass spectrometer Ultraflex II BRUKER (MALDI–TOF MS, Germany) equipped with a UV laser (Nd) in the mode of positive ions in a linear mode, using a reflectron. MS spectra were processed using software (FlexAnalysis 3.3 package, Bruker Daltonics, Germany). Using the Mascot program (peptide fingerprint option, www.matrixscience.com), homologs were searched in the NCBI database among proteins of all organisms. Candidate proteins with a score parameter above 83 in the NCBI database were considered to be reliably determined (*p* < 0.05). A molecular mass search was performed using Biotools 3.0 software (Bruker Daltonics, Germany).

*RNA isolation and RT-PCR Analysis*. Total RNA was isolated from 100 mg of a cell pellet using the Spectrum Plant Total RNA Kit (Sigma) according to the manufacturer’s protocol. Prior to RT-PCR, RNA was additionally purified from DNA by digestion with DNase I (Fermentas, Vilnius, Lithuania). RNA (1 mg) integrity was checked electrophoretically in a 1% agarose gel.

RT was done using Superscript III transcriptase (Invitrogen, United States). The reaction was carried out as recommended by the manufacturer; the reaction mixture contained 2 μg of total RNA and reverse PCR primers, which are listed in Table S1. The resulting cDNA was used as a PCR template.

RT-PCR products were analyzed by electrophoresis in a 1% agarose gel. For a quantitative analysis the One-Dscan program was used. A difference in the intensity of the bands greater than two times was considered significant.

*Statistics*. All experiments were performed with at least three independent biological experiments. The means for three analytical replicates and their standard errors (SE) are presented. Figures 2b, 4, and 5 show the results of a typical experiment. Comparisons of datapoints from different treatments with controls were performed using Tukey multiple pairwise comparison test. Differences of *p* < 0.05 were considered significant.

## 3. Results and Discussion

### 3.1. Assessing the Effects of ABA on Growth and Differentiation in Cell Suspension Cultures

**Figure 1:**
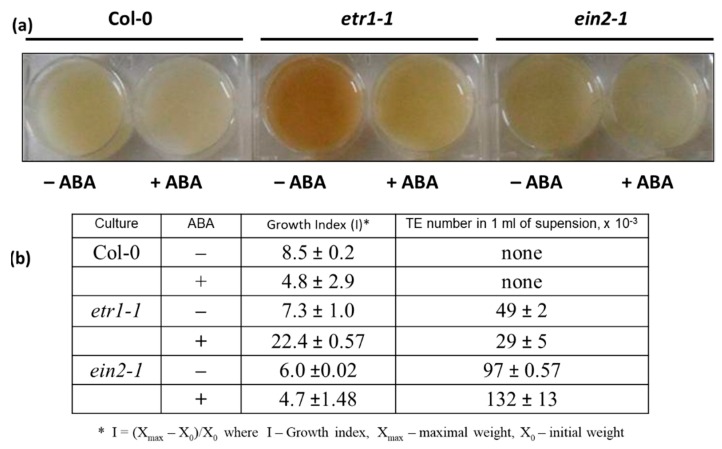
The effect of abscisic acid (ABA) on the phenotype (**a**) and growth indexes (Is) (**b**) of suspension cultures of *A. thaliana* Col-0, *etr1-1*, and *ein2-1*. Cell cultures of three genotypes were grown in the medium supplemented with 50 µM ABA (+ABA) for 10 days. (–ABA): control tissue culture not supplemented with ABA. At the end of sub-culturing, Is values and number of TEs were determined. Values presented are means of three independent experiments with five replicates in each ± SE (standard error). Data are significant at *p* < 0.05.

Actively proliferating cell cultures of wild-type *A. thaliana* (Col-0) and ethylene-insensitive mutants *etr1-1*, *ein2-1*, and *ctr1-1* have been established and extensively characterized. Mutant lines are visibly distinguished from Col-0. The cells of *ctr1* are significantly bigger than Col-0, and *etr1* and *ein2* contain many tracheary elements (TEs), i.e., a significant number of cells leave the cell cycle and follow the terminal differentiation. Moreover, in *etr1* apart from the TEs formation the cell death has occurred [6]. To assess the effects of ABA, Col-0, *etr1-1* and *ein2-1* cell cultures were used at day 5 following sub-cultivation, which is the stage when the most of the cells are in S-phase [6,33,38]. The cultures were inoculated into a fresh medium with 50 μM ABA. Then, growth indices (Is) and the number of TEs were determined. At the end of sub-cultivation, ABA-untreated cells of Col-0, *etr1-1* and *ein2-1* were similar in terms of Is (Figure 1). When cells were grown in ABA-supplemented medium, Col-0 cells retained 60%–70% viability, while the growth of *ein2-1* was not affected by ABA, and *etr1-1* behaved quite differently (Figure 1b). The *etr1-1* cells partly returned “back to life” which was reflected in a pronounce increase of I value, while the number of TEs was decreased (Figure 1b). These results were opposite to those observed in *ein2-1,* where the TE number was two to three times higher than in *etr1-1.* Therefore, we have suggested that both ETR1 and EIN2 are essential for normal cell cycle progression in cultivated cells.

A sequence of specific cellular events that lead to cell differentiation is required to form a mature TE and the study of this process in planta is difficult because differentiation occurs within complex tissues in the plant body. Opposite to intact plants, in vitro cell cultures that differentiate into a high percentage of TEs can be assessed at different time points after the induction of TE formation. Examples of successfully established in vitro TE systems include *A. thaliana* [39]. It was previously demonstrated that TE formation in a suspension of *Arabidopsis* cells can be induced by brassinosteroids [40] and inhibited by 2,4-dichlorophenoxyacetic acid (2,4-D). Auxins (especially synthetic 2,4-D, which is commonly used in a culture medium) are known to stimulate ethylene production. Since the *etr1-1* and *ein2-1* mutants are insensitive to ethylene, but TEs are actively formed in them, it can be assumed that the inhibition of TE formation in these cultures was ethylene- rather than 2,4-D-dependent. At the same time, one cannot ignore the fact that some ethylene-insensitive mutants are characterized by a high content of endogenous ABA, as shown, for example, in etiolated seedlings and intact *ein2-1* plants [4,23,27,28]. In addition, it has been shown that endogenous ABA in the absence of stress can accelerate growth by inhibiting ethylene synthesis [41,42].

### 3.2. Ethylene and ABA Production during the Sub-Cultivation Cycle and Effect of ABA on DNA Synthesis

Measurement of the content of endogenous ABA in the cell cultures during the subculture period showed that the amount of hormone in *etr1-1* and *ein2-1* lines tended to increase, while in Col-0 the amount of ABA did not change (Table 1). Based on these data, cells were treated with exogenous 25 μM ABA after 4-5 days of inoculation into a fresh medium, when the content of endogenous ABA was similar in all cultures.

This data should be considered together with the results on changes in ethylene biosynthesis during the period of subculture (Table 2). Apparently, if ethylene and ABA only affect each other’s synthesis in a cell culture, then in Col-0 with a relatively constant level of ABA (Table 1) ethylene synthesis should also remain at a constant level (Table 2). However, the data obtained do not confirm this assumption: the ethylene content in Col-0 cells decreased 17 times between the 4th and 10th day of subculture. On the 9th day of cultivation, in *etr1-1* and *ein2-1* lines, the ABA content was almost twice higher than at the day two (Table 2). At the same time, by the end of the sub-cultivation, *etr1-1* line synthesized twice as much ethylene, and the *ein2-1* line produced 12 times less ethylene than Col-0. Thus, if ethylene signaling is not interrupted, then ethylene negatively regulates ABA biosynthesis.

In dicots, such as the model species *A. thaliana*, leaves initiate at the flank of the meristem, and, in the initial phase, their growth is driven exclusively by cell proliferation. In older leaves, cells exit the cell cycle and begin to elongate, starting from the tip onward. This transition is marked by the onset of endo-reduplication, that is, a modified cell cycle in which DNA replication proceeds without mitosis, resulting in higher ploidy, such as 4C, and 8C. Cell proliferation is influenced by both genetic background and extracellular cues.

It is believed that in many cell types, ABA inhibits division and/or DNA synthesis at the G1/S without affecting other phases of the cell cycle [24]. Considering this data, we analyzed the effect of ABA on DNA synthesis, determining in vivo incorporation of thymidine analog BrdU into DNA. Cell cultures of all genotypes were treated for 1 hour with ABA (25 µM). One hour later, BrdU (150 µM) was added, and cells were incubated with these two chemicals for two hours. DNA was isolated, denatured and immobilized on a nitrocellulose membrane. The BrdU incorporation into DNA was evaluated immunologically via staining with anti-BrdU antibodies (1:2000 dilution) [31].

**Figure 2:**
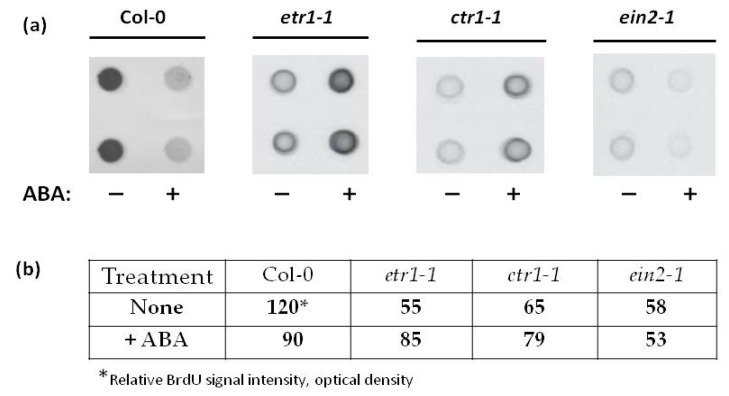
ABA (25 µM) affects 5-bromo-2-deoxyuridine (BrdU) incorporation into the DNA of Col-0, *etr1-1*, *ctr1-1*, and *ein2-1* cell lines. Genomic DNA (2 μg) was isolated from cultured cells and denatured. Single-stranded DNA was applied to Hybond-C using a Bio-Dot SF Microfiltration apparatus (**a**). BrdU incorporation into DNA was quantified (**b**) with monoclonal anti-BrdU antibodies (1:2000 dilution). For visualization, anti-mouse antibodies (dilution 1:7000) conjugated with horseradish peroxidase were used. Numbers in (**b**) correspond to relative BrdU signal intensities quantified as optical density of dot-blots.

In Col-0 culture in the middle of the exponential growth phase, a short ABA treatment caused a decrease in BrdU incorporation into DNA (Figure 2). This can be interpreted as a negative effect of ABA on DNA synthesis, since, in all cell lines used in these experiments, the content of endogenous ABA is similar (Table 2). These results are consistent with the previously reported data [23].

Unlike in Col-0, in ABA-treated *etr1-1* cell line the BrdU incorporation into the DNA increased significantly, while no ABA effect was found in *ctr1-1* and *ein2-1* mutant cell lines (Figure 2). Therefore, in in vitro cultured cells, ethylene insensitivity can affect ABA signaling, which normally leads to inhibition of DNA synthesis.

Data on the ABA effect on DNA synthesis should be considered in parallel with the results of determining of mitotic activity. For such a comparison, we will use the data of Stepanchenko et al. [6] that demonstrate that, under standard conditions, the DNA synthesis in the exponential growth phase correlates (r = 0.98) with the mitotic activity of suspension cultures.

When the ethylene signal transduction pathway functions normally, as in Col-0, ABA inhibited DNA synthesis (G1/S transition) and did not affect the value of the mitotic index (5.4%) (Figure 2). Tracheary elements appeared massively only in *etr1-1* and *ein2-1* suspensions, i.e., a certain fraction of cells followed the terminal differentiation (TE formation), which probably affected the mitotic activity of *etr1-1* and *ein2-1* cells. Their mitotic indexes were halved to 2.8% and 2.7%, respectively. In this case, exogenous ABA, in the absence of ethylene signal perception, stimulated DNA synthesis in *etr1-1* but not in *ein2-1* cultures, where the ethylene signal is perceived but not transferred (Figure 2).

A significant part of *ctr1-1* cells was preoccupied by DNA endoreduplication and, probably, was delayed in the G2 phase. Exogenous ABA did not affect DNA synthesis in *ctr1-1* cells, but prevented the transition of cells to endo-reduplication, as can be seen from the increase in the mitotic index from 3.5% to 4.3%.

### 3.3. ABA Affects Soluble Proteins Phosphorylaion in Col-0 and Ethylene-Insensitive Mutant Cell Cultures

Despite the unique fate of cells in *etr1-2*, *ctr1-1*, and *ein2-1* cultures, all three cell lines can be stably maintained in vitro, which indicates the unaffected functioning of cyclin-dependent protein kinases that ensure progression through the cell cycle. We focused on the search for proteins whose functions do not duplicate the steps of the universal cyclin-dependent cell cycle machine.

**Figure 3:**
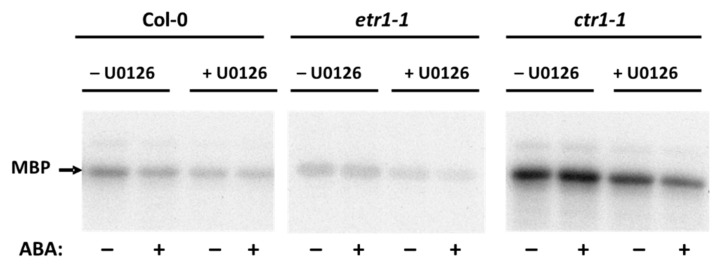
Effect of U0126 protein kinase kinase (MKK) inhibitor on ABA-dependent MBP-phosphorylation of the soluble proteins isolated from Col-0, *etr1-1*, and *ctr1-1* cultures. Soluble proteins were isolated from ABA/U0126-untreated cells and treated with both substances. Myelin basic protein (MBP) phosphorylation was tested in vitro with [γ-^32^P]ATP. Radioautographic image is presented.

Extensive research in *Arabidopsis* led to the identification of key elements in the ethylene and ABA signaling cascade. Since mitogen-activated protein kinases (MAPKs) are components of the ethylene and ABA signaling [43,44,45,46,47,48,49], we assumed that the effect of these phytohormones could be associated with changes in the enzymatic activity of these protein kinases. As the MKK9-MPK3/MPK6 cascade is involved in not only in ethylene biosynthesis, but also ethylene signaling acting downstream of CTR1 [46] to make sure that mitogen-activated protein kinase kinase (MKK) can be found among ABA-regulated phosphoproteins, the cells were first treated with a specific MKK inhibitor, U0126, and then with ABA. Proteins isolated from U0126-treated cells demonstrated a decrease in the phosphorylation level of MBP, indicating the presence of MPK, the specific substrate for MKK (Figure 3).

### 3.4. The Effect of ABA on Transcription of Individual MPK Genes

It is known that, in tobacco BY-2 cell culture, high activity of mitotic cyclin-dependent protein kinases is required for the phosphorylation of nucleus- and phragmoplast-localized protein kinase 1 (NPK1) and NPK1-activating kinesin-like protein 1 (NACK1), and that this phosphorylation inhibits the MAPK cascade, thereby preventing M phase cells from initiating cytokinesis before anaphase [50]. If ABA is able to regulate proliferation, can this effect be associated with changes in MPK activity, especially since there is evidence of ABA regulation of MPK activity [49]? To answer this question, all the cell cultures in the phase of exponential growth were incubated with 25 μM ABA for three hours (Table 3). The concentration of exogenous ABA and the exposure time were chosen to avoid affecting the ethylene biosynthesis.

As it was demonstrated earlier, the application of exogenous ABA resulted in the regulation of the transcription, protein accumulation and activation of MPKs [51,52,53,54,55,56,57,58]. In order to analyze the effect of ABA on the expression of *MPK*s, we selected genes encoding MPKs belonging to the three MPK groups, namely, MPK3 and MPK6 belonging to group A, MPK4 and MPK5 to group B, and MPK1 and MPK2 to group C [58].

**Figure 4:**
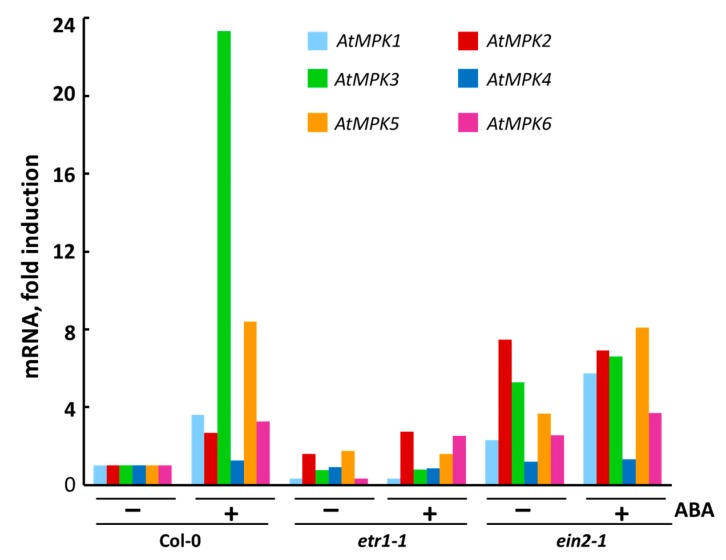
Expression of individual genes coding for *MPK*s in Col-0, *etr1-1*, and *ein2-1* cultured cells after 3 h treatment with ABA. mRNA levels in cultured Col-0, *etr1-1* and *ein2-1* cells were examined by RT-PCR. For RT-PCR analysis, At*ACT2* was used as a reference gene. Data on a typical experiment are presented.

Using RT-PCR, we found that during the exponential growth phase of Col-0, *etr1-1*, and *ein2-1* cultures, the constitutive level of *MPK4* transcription was higher than that of other *MPK*s genes and depended on the treatment with exogenous ABA in Col-0 only (Figure 4). On the contrary, the *MPK1* and *MPK2* genes were transcribed at an extremely low level, which coincides with the literature data [59].

After ABA treatment, *MPK1* expression increased in Col-0 and *ein2-1*, while *MPK2* transcription increased significantly in both Col-0 and *etr1-1*, but did not change in *ein2-1*. Although the MPK1 and MPK2 proteins are 87% identical, their sensitivity to ABA can vary. It cannot be ruled out that, in the same interval of treatment with exogenous ABA, the maximum activation of expression of *MPK1* occurs earlier than in the case of *MRK2*. Therefore, it can be assumed that MPK1 in proliferating cells is associated with ABA signal transduction. This assumption is important, given that there are known differences in the time-dependent activation of MPK1 and MPK2 [59].

In the Col-0 and *ein2-1* cell lines, *MPK5* expression was doubled after treatment with ABA, whereas, in *etr1-1,* ABA had no effect on *MPK5* expression. *MPK3* expression was highly induced in ABA-treated Col-0, while in *etr1-1* and *ein2-1* it was extremely low and ABA-independent. In ABA-treated Col-0, *MPK6* expression was more than three times higher compared to the control cell culture. On the contrary, in *ein2-1*, *MPK6* transcription was ABA independent, since the activation was only 1.4 times higher after ABA treatment. However, in *etr1-1* cells, ABA treatment significantly affected *MPK6* expression (more than seven times) (Figure 4). Since *etr1-1* does not perceive ethylene due to a mutation in the ethylene receptor ETR1, ABA should be considered responsible for the detected change in the level of MPK6 expression in *etr1-1*.

### 3.5. The Effect of ABA on Transcription of Individual SnRK2 Genes

It is currently accepted that, in plants, signaling events involving ABA begin with the biosynthesis of the hormone, their long-distance transport, followed by the perception of ABA by their receptors and the initiation of the ABA signal transduction pathway [8,9,12]. Protein phosphatases PP2C (Group A) and the SnRK2 protein kinases are the key components of the ABA-signaling pathway, initiated after the binding of ABA to the receptors.

In *Arabidopsis*, there are 10 protein kinases of the SnRK2 family, which are divided into three subfamilies [60]. However, only SnRK2.2/2.3/2.6/2.7/2.8, belonging to subfamily III, were activated by ABA [3,11,61,62] with the highest sensitivity to ABA found for SnRK2.2/2.3/2.6 [3,63,64].

**Figure 5:**
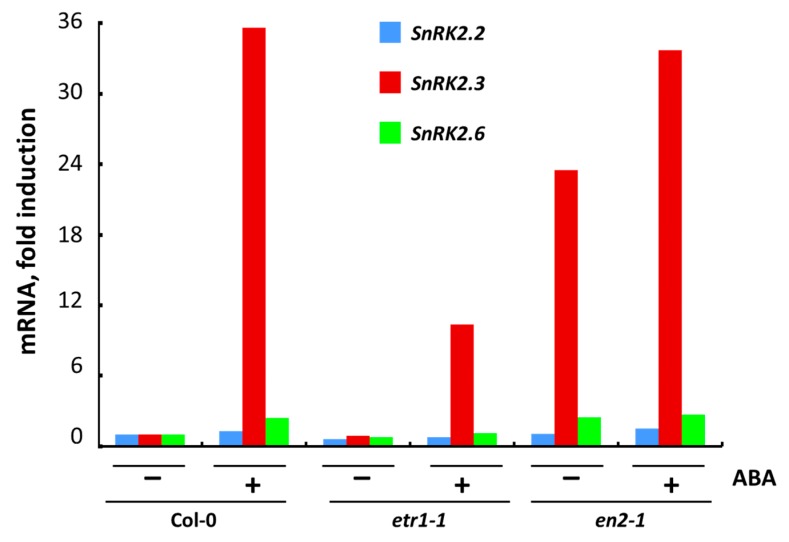
Expression of individual genes coding for *SnRK2*s in Col-0, *etr1-1* and *ein2-1* cultures after 3 h treatment with ABA, estimated by RT-PCR with At*ACT2* as a reference gene. Results of a typical experiment are presented.

We studied the effect of a three-hour treatment with exogenous ABA on the expression of the *SnRK2.2, SnRK2.3* and *SnRK2.6* in Col-0 and ethylene-insensitive mutant cell cultures (Figure 5). We found that in all untreated cultures the level of *SnRK2.3* expression was strongly ABA-regulated.

In *ein2-1*, a high level of expression of all *SnRK2* in the control may indicate that a point mutation in the *EIN2* gene leads to a constitutive functioning of the ABA signaling pathway. Apparently, this can be associated with the hypersensitivity of *ein2* mutants to ABA [27,28,65]. Taking into account that the endogenous ABA content is similar in Col-0, *etr1-1* and *ein2-1* cells, the observed changes in the expression of *SnRK2.2/2.3/2.6* were probably due to exogenous ABA application.

In the literature, it has been established that *etr1-1* and *ein2-1* display an increased sensitivity to ABA in comparison to Col-0, at least during seed germination and early seedling growth [5,27]. To what extent is the physiological response to ABA consistent with the function of the ABA signal transduction pathway? Our analysis of *SnRK2.2/2.3/2.6* expression suggests that, in unstressed cells, the change in the fate of cultured cells—active proliferation or terminal differentiation—cannot be explained by only the activity of ABA signaling pathway. It must be also dependent on the functional activity of both ethylene receptors and the components of the ethylene signal transduction pathway.

### 3.6. The effect of ABA on MAPK Activity in Col-0 and Ethylene-Insensitive Mutant Cell Cultures

Since the preferable MPKs’ in vitro substrate is myelin basic protein (MBP), we decided to use isoelectric focusing under native conditions. In order to enrich protein fractions with MPKs we used isoelectric focusing under native conditions. Cytosolic proteins isolated from cells were fractionated using MicroRotofor Liquid-Phase IEF Cell, which allows the enrichment of low-abundance proteins [66] to enhance the results from downstream applications.

**Figure 6:**
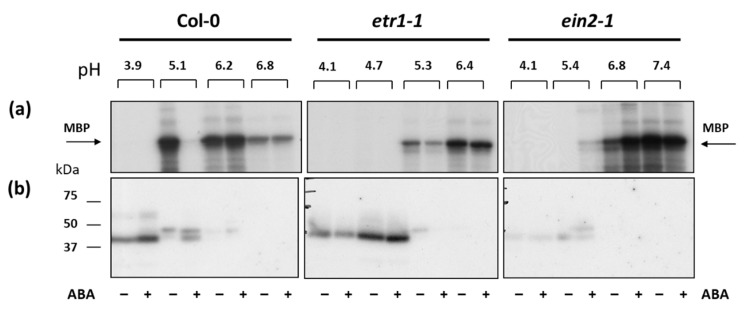
The effect of ABA (25 µM) on the MBP phosphorylation in vitro (**a**) and in gel (in situ) (**b**) in protein fractions enriched with low-abundance proteins using a MicroRotofor IEF Cell. Radioautographic image is presented.

After focusing, 10 protein fractions with a pH range from 5.0 to 7.0 were obtained for proteins isolated from untreated and ABA-treated cell cultures of each genotype (Figure 6). In Col-0, in the fraction with pH 5.1, the MBP phosphorylation, a preferable MPKs in vitro substrate, decreased, and in the fraction with pH 6.2 it increased after treatment with ABA. In *etr1-1*, the level of ABA-dependent MBP phosphorylation decreased in both fractions, and, in *ein2-1*, the level of MBP phosphorylation was increased after ABA treatment in both fractions. Thus, we focused on these fractions.

In order to characterize further the proteins associated with MBP-kinase activity, proteins were electrophoresed and renaturated within the gel prior to phosphorylation with radiolabeled ATP (a typical autoradiograph is shown in Figure 6b). MBP-phosphorylating activity was associated with proteins of Mr∼41 kDa and Mr∼45 kDa in both Col-0 and mutant cell lines. Phosphorylation of 41-kDa polypeptide was promoted by ABA treatment in Col-0 (fractions with pH 5.1–5.4), while in *ein2-1* the 45-kDa MBP-kinase was activated. This suggests that, depending on the functional activity of the ethylene signaling pathway, different individual MPKs are activated in response to ABA.

### 3.7. Identification of MAPKs and Their Putative Substrates with MALDI-TOF MS

**Figure 7:**
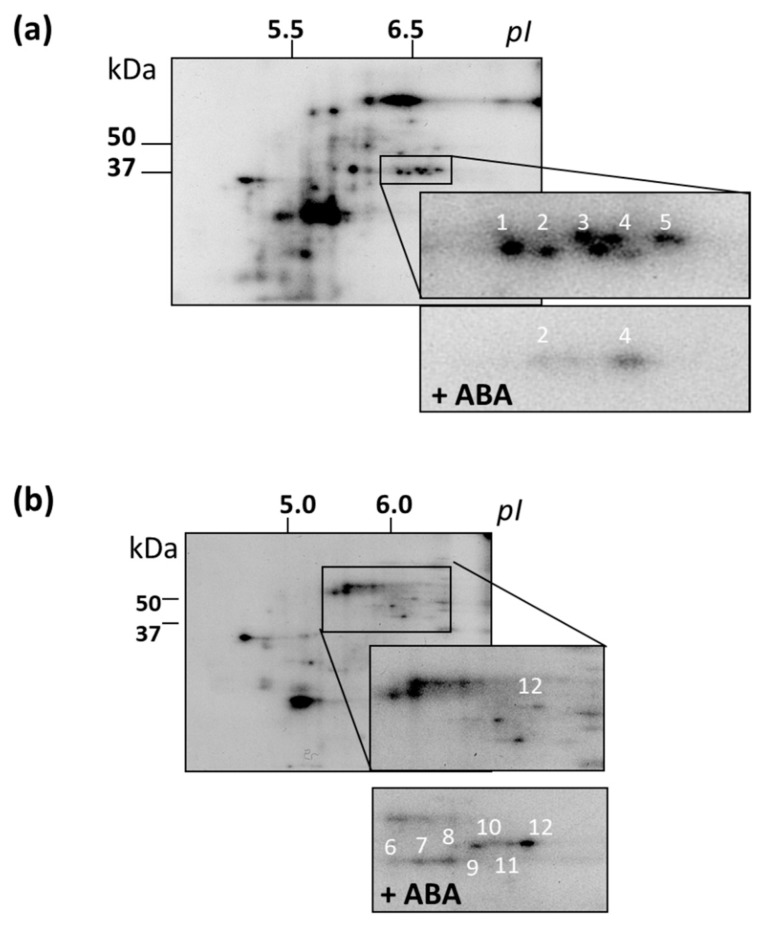
Radioautographic images of 2D-gels of the ABA-dependent phosphorylated proteins separated and enriched in the MicroRotofor IEF Cell. Fractions with pH 5.2 – 5.4 (**a**) and 6.2 – 6.8 (**b**) were used for 2D-gels (7 cm strips, pH 4.0–7.0). The proteins identified by MALDI-TF MS are numbered.

To obtain a more detailed picture of the effect of ABA on protein kinase activity, we performed in vitro phosphorylation reaction (without an exogenous substrate) with protein fractions which exhibited MPK activity. For each genotype, groups of phosphoproteins specific for both ABA-treated and control cells were identified. For most proteins, their phosphorylation level significantly decreased after ABA treatment (Figure 7).

For all cell lines, we selected 12 individual phosphoproteins for identification by finger prints. The following proteins were identified: NADP-dependent oxidoreductase (spot 1), annexin2 (ANNAT2, spot 2), aldo/ketoreductase (spot 3), PYL8 (spot 4), glyceraldehyde-3-phosphate dehydrogenase (spot 5), KRP4 (Kip-related protein, spot 6), two enolases (spots 7, 9), ATP synthase β-subunit (spot 8), translation initiation factor 4A (eF4A, spot10), MPK11 (spot 11), transcription factor SCL9 (from Scarecrow-like 9, spot 12) (Table 4 and Figure 7). The phosphorylation of proteins from spots 1, 2, 3, and 5 was lower in ABA-treated cells, whereas the phosphorylation level of the rest of proteins was higher. We analyzed the sequences of these proteins in the NetPhos and NetPhosK databases, and found that in some of the identified proteins, namely, in KRP4 (spot 6), eIF4A (spot 10), and SCL9 (spot 12), there are PX(S/T)P motifs present. These motifs could be phosphorylated by MPKs. In other words, it is quite possible that proteins with these motifs are potential substrates of MPKs. However, it cannot be ruled out that other protein kinases could carry out ABA-dependent phosphorylation of these proteins. For example, it has been shown that some substrates of MPK3/6 can be phosphorylated by CaM kinase II (Ca^2+^/Calmodulin dependent kinase II), PKC (Protein kinase C), and MAPKAPK2 (MAPK activated protein kinase 2) [67].

The protein MPK11 (spot 11) is one of the most interesting proteins out of all identified. The closest homolog of MPK11 is MPK4 [68]. To further confirm the identification of the phosphorylated form of MPK11, we concentrated the trypsin-digested protein spot 11 using magnetic beads coated with TiO_2,_ then applied a strong anion exchanger (Polysulfoethyl A SCX) and subjected the sample to LC-ESI MS/MS. Thus, MRK11 was reliably determined by two different MS procedures.

Based on the literature data for identified proteins, their potential role in cell division and/or differentiation can be suggested. SCL9 transcription factor is of a special interest, since in *Arabidopsis* roots SCRs are shown to accumulate in the quiescent center and regulate asymmetric cell division [69,70,71]. Ethylene treatment increases the number of cells in the quiescent center of the wild-type *Arabidopsis* root and *ctr1-1* [20]. Since, in our work, ABA caused an increase in DNA synthesis in *etr1-1*, both ethylene and ABA could be considered as signals for SCL9 phosphorylation.

The most often proposed scenario of stress-induced cell cycle inhibition assumes transcriptional upregulation of genes for cell cycle inhibitors such as cyclin-dependent kinase inhibitor (ICK)/KIP-related protein (KRP). These inhibitors are thought to transiently arrest cell proliferation by inhibiting CYCLIN-DEPENDENT KINASE A (CDKA)/cyclin complexes. CDKA activity, which is a main driver of the cell cycle progression, can also be reduced via targeted degradation of cyclins and/or inhibitory phosphorylation. ABA has been demonstrated to affect the expression of *ICK/KRP*. KRP4 protein, identified in our experiments, is one of the seven *Arabidopsis* KRP proteins. Previously, in a two-hybrid yeast system, it was shown that cyclin–cyclin-dependent protein kinase (CDK) complexes can interact with all *Arabidopsis* KRPs [71]. The phosphorylation of KRP is known to affect its binding to CDKA, and therefore affect the development of G1/S/G2 [72]. On the other hand, in the cells that underwent endoreduplication, the kinase activity of CDKB is decreased, and KRP can bind and inhibit CDKA [71]. In *Arabidopsis*, overexpression of KRP1 results in a reduction in both the number of endocycles and trichome cell size [73]. The peak of *KRP4* expression occurs in the G2 phase [74], and the KRP4 protein is responsible for the correct progression via the S phase [75].

In eukaryotes, the phosphorylation of translation initiation factor eIF4A leads to the increased assembly of polysomes and, as a result, to up-regulation of protein synthesis. Mammalian eIF4A is a substrate of MAPKAPK1, which is phosphorylated by ERK1 and JNK [76]. In mammals, MAPKAPK2 regulates the S phase as well as G2/M in response to UV-C [77]. As far as we know, MAPKAPK1/2 has not yet been found in plants, but in *Arabidopsis* there are proteins containing PPR repeats (pentatricopeptide repeat-containing), as well as protein kinases with calmodulin-like domains, which have 38% and 37% homology with MAPKAPK1/2, respectively.

Of particular interest is the PYL8, a member of the ABA receptors family. We have shown that PYL8 is phosphorylated in *Arabidopsis* cells. Taking into account the mechanism of ABA signaling, the question arises, as to which state—phosphorylated or dephosphorylated—should the ABA receptor be in, in order to both bind ABA and initiate SnRK2 protein kinase cascade to enable signal transduction? Based on MS analysis data, we can assume that ABA-dependent protein phosphorylation is associated with the G1 and S phases. Our data are consistent with the *Arabidopsis* transcriptome, which shows that transcription of the genes encoding the proteins discussed above is also associated with cell division [78].

## 4. Conclusion

Here, we report the results of the initial study on a mechanism of control of plant cell proliferation and differentiation via interaction of ethylene and ABA signal transduction pathways. The cross-talk of ethylene and ABA signaling routes have been demonstrated upon seed germination [3,4,79], stomata cells movement [78,79], and changes in root growth, including the formation of lateral roots under heat stress [80,81]. However, based on these data, it is difficult to specify the effect of ethylene and ABA on cell division. At the same time, ethylene rather than auxin, is recognized as a positive regulator of cell proliferation. Ethylene enhances cell division in the quiescent center, which acts as a source of stem cells in the *A. thaliana* seedling root [20]. Although ABA is often considered as a growth inhibitor hormone, there is an evidence that ABA at low concentrations can activate growth [27,28].

Using suspension tissue cultures of *A. thaliana* Col-0, as well as ethylene-insensitive mutants *etr1-1*, *ctr1-1*, and *ein2-1*, we were able to show that ethylene and ABA do not influence the biosynthesis of each other (Table 2; Table 3). Indeed, a short-term treatment with exogenous ABA did not significantly enhance ethylene emission. During cell cultivation, however, Col-0 cells evolved ethylene in quantities sufficient for continuous cell division. It seems likely that the interaction of ethylene and ABA is associated with the regulation of the target genes’ expression.

The binding of exogenous ABA to the corresponding receptor(s) leads to the interaction of the protein phosphatase PP2C with the hormone-receptor complex and to the inactivation of SnRK2 kinases. As a result, protein kinases SnRK2s are released from PP2C inhibition. Being autoactivated, SnRK2s execute ABA-dependent phosphorylation of their targets. Among them, there may be MPKs that are involved in ABA-mediated reduction in proliferation by activation of KRP4, as we identified in this work.

In cell cultures, ethylene is a mitogen, but the mechanism of its action has been poorly understood. Downstream of ethylene receptors, the MAP kinase module with AtMPK3/6 operates [43]. In response to ethylene, expression of PP2C genes is enhanced [82] because PP2C subgroup B and some representatives of subgroup A have domains that ensure their binding to MPK, including MPK6 [82]. The binding of PP2C to MPK6 leads to a decrease in MPK6 activity, and, as a result, autoregulation of ethylene biosynthesis can occur to allow cells to proliferate.

**Figure 8:**
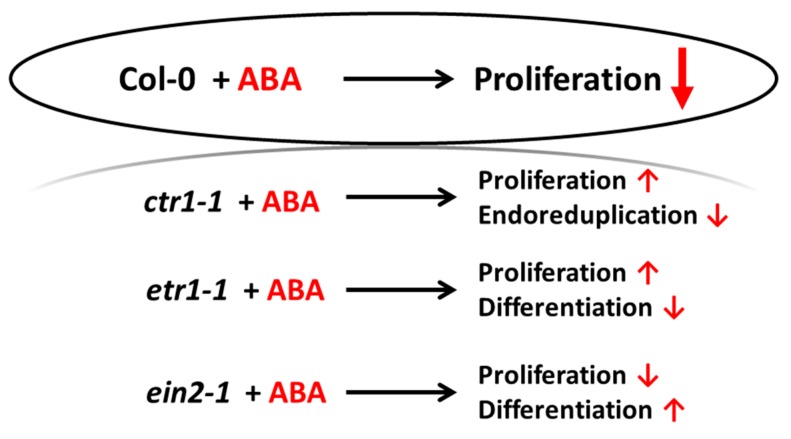
Schematic representation of the cellular events occurring in cultured *Arabidopsis* cells treated with ABA.

This study provides the evidence for the ethylene–ABA interaction while regulating cell division and differentiation as follows (Figure 8). When the ethylene signal transduction pathway is functionally active (Col-0), the cells actively proliferate, and exogenous ABA performs its function as an inhibitor of DNA synthesis and division. If ethylene signal is not perceived (*etr1-1*), then, in addition to differentiation (TE formation), cell death can occur, and exogenous ABA rescues the cells, increasing proliferation. If the ethylene signal is perceived, but not transduced (*ein2-1*), then differentiation takes place, which is enhanced by exogenous ABA, while cell proliferation is reduced. When the transduction chain is constitutively active, the cells begin to exit the cell cycle and proceed to endoreduplication (*ctr1-1*). In this case, exogenous ABA promotes the reactivation of cell division.

Thus, the perception and transduction of the ethylene signal determines the ratio of cell proliferation and differentiation/death, which can be adjusted by ABA. Apparently, in the absence of ethylene perception, the underlying mechanism(s) is different from those accompanying aging.

## Figures and Tables

**Table 1:** The endogenous ABA content (ng/g FW) in suspension tissue cultures Col-0, *etr1* and *ein2-1* during sub-cultivation. Values presented are means of three independent experiments with five replicates in each ± SE (standard error). Data are significant at *p* < 0.05.

Days	Col-0	*etr1-1*	*ein2-1*
2	4.3 ± 0.21	2.9 ± 0.15	2.4 ± 0.12
4	4.0 ± 0.20	3.5 ± 0.18	2.6 ± 0.13
6	3.6 ± 0.18	4.4 ± 0.22	3.0 ± 0.15
9	3.3 ± 0.16	5.6 ± 0.28	4.8 ± 0.24

**Table 2:** The effect of exogenous ABA (50 μM) on ethylene biosynthesis in suspension tissue cultures Col-0 0, *etr1-1* and *ein2-1* during sub-cultivation. Values presented are means of three independent experiments with five replicates in each ± SE (standard error). Data are significant at *p* < 0.05.

Cultivation, Days	ABA	Ethylene Evolution, nL g^−1^ FW h^−1^		
		Col-0	*etr1-1*	*ein2-1*
4	−	595 ± 15	281 ± 4	18 ± 3
	+	482 ± 27	288 ± 18	31 ± 1
6	−	323 ± 7	268 ± 1	12 ± 2
	+	464 ± 7	266 ± 23	22 ± 1
10	−	34 ± 5	82 ± 45	2.7 ± 0.04
	+	238 ± 22	66 ± 27	3.5 ± 0.25

**Table 3:** Ethylene evolution (nL/g FW/h) by cell cultures Col-0, *etr1-1* and *ctr1-1* treated with ABA (25 µM) for three hours. Values presented are means of three independent experiments with five replicates in each ± SE (standard error). Data are significant at *p* < 0.05.

Culture	Untreated	ABA Treated
Col-0	280 ± 6	250 ± 12
*etr1-1*	160 ± 6	150 ± 8
*ctr1-1*	130 ± 8	200 ± 20

**Table 4:** List of phosphorylated proteins identified by MALDI-TOF MS *.

Protein	*pI*	M_r_, Da
NADP-dependent oxidoreductase (spot 1)	5.8	38133
Annexin (ANNAT2) (spot 2)	5.76	36243
Aldo/keto reductase (spot 3)	5.92	37900
Pyrabactin resistance-like 8 (PYL8) (spot 4)	6.07	21397
Glyceraldehyde-3-phophate-dehydrogenase (spot 5)	6.67	36913
Kip-related protein 4 (KRP4) (spot 6)	9.61	31699
Enolase (spot 7)	5.79	51477
ATP synthase β-subunit (spot 8)	6.18	59713
Bifunctional enolase (spot 9)	5.54	47689
Translation initiation factor 4A (eF4A), (spot 10)	5.47	46674
Mitogen-activated protein kinase11 (MPK11) (spot 11)	5.54	47689
Scarecrow-like 9 (SCL9) (spot 12)	5.66	53344

* The entire list of protein characteristics is available in Supplementary Table S2.

## Supplementary Materials

Supplementary materials can be accessed at https://www.mdpi.com/2075-1729/10/2/15/s1.
