# Supplementary material for: Coupling of Cell Division and Differentiation in Arabidopsis thaliana Cultured Cells with Interaction of Ethylene and ABA Signaling Pathways
Source: Life (Basel). 2020 Feb 10;10(2):15. doi: 10.3390/life10020015 (PMC7175341; doi:10.3390/life10020015)
Supplement: Supplementary file 1 [file life-10-00015-s001.pdf]

Supplementary Table S1. Sequences of oligonucleotide primers used for RT-PCR analysis.

Gene name	Gene ID	Forward or reverse primer (5' - 3') (F or R)
AtMPK1	AT1G10210	5'-ATAGGATCGATGCGTTGAGG-3'
		5'-TCGGTACCAACGAGTCACAA-3'
AtMPK2	AT1G59580	5'-TCTGGTCAGTCGGTTGCATA-3'
		5'-TCATCTCTGCTCCCAAATCC-3'
AtMPK3	AT3G45640	5'-ATGAACACCGGCGGTGGC-3'
		5'-ATGAACACCGGCGGTGGC-3'
AtMPK4	AT4G01370	5'-CTGCTGCACTTCTAACCG-3'
		5'-GGAGCTCGATACCAACGTGT-3'
AtMPK5	AT4G11330	5'-GACTTTGGTTTGGCAAGGAC-3'
		5'-GACGGATCCTCAAAATGGAA-3'
AtMPK6	AT2G43790	5'-TCTCCTCCTGAACGCAAACT-3'
		5'-GATGGATTGGCGAGGATAAG-3'
AtSnRK2.2	AT3G50500	5′-CCGATTATGCCGATTGATTT-3'
		5′-TATCGGTCATGAGACGAGCA-3′
AtSnRK2.3	AT5G66880	5′-GCAATGCAGGACGGTTTAGT-3'
		5′-GAGCAGGACTTCCATCCAAC-3'
AtSnRK2.6	AT4G33950	5′-AACTTTTCGAGCGAATCTGC-3'
		5′-GCCGGGCTACCATCTAATAAC-3'
AtACT2	AT3G18780	5'-GCCGGACTTACCGTTGTATGTACCGTCC-3'
		5'-ACAATGGAACTGGAATGGTGAAGGCTGG-3'

Supplementary Table S2.. List of phosphorylated proteins identified by MALDI-TOF MS.

PROTEIN	*PI*	M_R, DA	PEPTIDE M_R, DA	PEPTIDE AMINO ACID SEQUENCE
**ANNEXIN (ANNAT2) (SPOT 2)**	5.76	36243	928.5612; 965.5169<br>982.4814; 1096.5354<br>1117.5187; 158.7420<br>1165.5585; 181.5619<br>1427.7725<br>1656.8458	**K.HFEKVLR.L; R.IKEEYQR.R;<br>K.ELSSDFER.A; K.AFSGWGTNEK.L;<br>K.SYSDDDFIR.I; K.LLLPLVSTFR.Y;<br>K.MGTDEWGLTR.V; K.MGTDEWGLTR.V;<br>R.AVMLWTLDPPER.D;<br>K.SIEEDVAQHTSGDLR.K**
**NADP-DEPENDENT OXIDOREDUCTASE (SPOT 1)**	5.8	38133	1223.5746; 238.7157;<br>1270.7541; 417.7319;<br>1600.8006<br>2234.1640<br>2527.1566<br>2861.3954	**K.FGFDDAFNYK.E; K.APEALVGLFHGK.N;<br>K.FLEFVLPHIR.E; R.IQGFVVSDFYDK.Y<br>K.MLDAVLVNMNMHGR.I;<br>K.EGETVYVSAASGAVGQLVGQLAK.M;<br>K.DYVSGFPTESDFDFTTTTVELR.V;<br>R.MGKPDPSTAALAQAYTPGQPIQGYGVSR.I**
**ALDO/KETO REDUCTASE (SPOT 3)**	5.92	37900	810.3996; 835.3664;<br>971.5091; 1100.5677;<br>213.6559; 1229.7031;<br>1367.7360; 374.6794;<br>1444.8326;<br>1679.8475	**R.GFFASGPK.L; R.GDPEYVR.A;<br>K.FGISYAEGK.R; R.YSNMIPTFK.N;<br>K.NAETPPLSAWK.A; R.VPIEITMGELK.K;<br>K.YIGLSEASASTIR.R; R.DVEEEIIPTCR.E;<br>R.ELGIGIVAYSPLGR.G;<br>R.GDPEYVRAACEASLK.R**
**PYRABACTIN RESISTANCE-LIKE 8 (PYL8) (SPOT 4)**	6.07	21397	873.4504; 1187.6611<br>1360.7544;<br>2266.1177;<br>2349.1974	**R.ALGQISER.L; K.VQTSSGEKPVR.E;<br>K.MELIDAAFPLLK.G;<br>K.NVIIWGNHSSSQYPDVNHAK.V;<br>R.ELVKDDAWLDGEFISTVQQR.G**
**GLYCERALDEHYDE-3-PHOPHATE-DEHYDROGENASE (SPOT 5)**	6.67	36913	910.4607; 1677.0152;<br>1734.8526;<br>1761.8269;<br>2033.0968;<br>2172.0374;<br>2923.4282	**K.AATYDEIK.K; K.TLLFGEKPVTVFGIR.N<br>K.DAPMFVVGVNEHEYK.S;<br>K.LVSWYDNEWGYSSR.V;<br>R.FGIVEGLMTTVHSITATQK.T;<br>K.GILGYTEDDVVSTDFVGDNR.S;<br>R.NPEDIPWGEAGADFVVESTGVFTDKDK.A**
**TRANSLATION INITIATION FACTOR 4A (EF4A), (SPOT 10)**	5.47	46674	876.4314; 976.5455;<br>1004.5385; 104.6503;<br>1114.6505; 135.5559;<br>1173.6530;<br>1348.6169;<br>1401.7292;<br>1555.7149;<br>1589.8197;<br>1784.7525;<br>1827.9360	**K.VDWLTDK.M; K.GVAINFVTR.D;<br>R.KVDWLTDK.M; R.KGVAINFVTR.D;<br>R.VLITTDLLAR.G; R.TWPICCEGR.K;<br>K.RDELTLEGIK.Q;<br>M.AGSAPEGTQFDAR.Q;<br>K.GLDVIQQAQSGTGK.T;<br>K.MFVLDEADEMLSR.G;<br>K.DQIYDIFQLLPPK.I;<br>R.DHTVSATHGDMDQNTR.D;<br>R.GIYAYGFEKPSAIQQR.G**
**BIFUNCTIONA ENOLASE (SPOT 9)**	5.54	47689	765.3841; 806.4558;<br>1101.5309; 229.6311;<br>2005.0843;<br>2324.0910	**R.QIFDSR.G; K.YNQLLR.I;<br>K.AGWGVMTSHR.S; K.KAGWGVMTSHR.S;<br>K.VTAAVPSGASTGIYEALELR.D;<br>K.YGQDATNVGDEGGFAPNIQENK.E**
**MITOGEN-ACTIVATED PROTEIN KINASE11 (MPK11)**	5.54	47689	765.3837; 806.4539;<br>900.4698; 1101.5264;<br>1117.5288; 212.6180;	**R.QIFDSR.G; K.YNQLLR.I;<br>K.TYDLNFK.E; K.AGWGVMTSHR.S;<br>K.AGWGVMTSHR.S; K.MGVEVYHHLK.S;**

(SPOT 11)			1549.8799;	K.AVGNVNNIIGPALIGK.D;
			1573.8236;	K.VNQIGSVTESIEAVK.M;
			1852.9397;	K.LAMQEFMILPVGAASFK.E;
			1896.9037;	R.IEEELGSEAIYAGVNFR.K;
			2005.0633;	K.VTAAVPSGASTGIYEALELR.D;
			3000.4809	K.SFVAEYPIVSIEDPFDQDDWEHYAK.M
SCARECROW-LIKE 9 (SCL9)	5.66	53344	906.4796; 994.5125;	R.WVFPETK.A; R.DSAAVFAWK.G;
(SPOT 12)			998.6149; 1025.5364;	K.AGIIVLAEGR.L; R.HSLPDGLMR.A;
			1026.4678; 071.6515;	K.DMSQADFGR.L; R.ITIKPQTDR.W;
			1129.5799; 137.5915;	K.DIIMVDHMR.K; R.TEFGPSQPFK.G;
			1475.7779;	R.LVGVSEETTTGVKR.L;
			1771.8082;	K.GETLQEYWWCTER.A;
			2361.1747	K.DQSDYVSIPIEGPYKPPHYR.Y
KIP-RELATED PROTEIN 4	9.61	31699	738.3476; 815.3790;	R.FEWEK.V; R.GADGGPWR.R;
(KRP4)			1205.6408; 1373.6453;	K.VGTLGLDMMLR.T;
(SPOT 6)			1418.7262;	R.SHIWTDTDKDR.T;
			1438.7450	K.EAGFLNAVDEVVR.T; K.FGFEVNTLRPMK.Y
ENOLASE	5.79	51477	765.3763; 1101.5415;	R.QIFDSR.G; K.AGWGVMTSHR.S;
(SPOT 7)			1174.6890;	K.AGAVVSGIPLYK.H;
			1859.8669;	K.VVIGMDVAASEFYSEDK.T;
			1896.9358;	R.IEEELGSEAIYAGVNFR.K;
			2005.0788;	K.VTAAVPSGASTGIYEALELR.D;
			2252.1494;	R.SGETEDTFIADLAVGLSTGQIK.T;
			2324.0840;	K.YGQDATNVGDEGGFAPNIQENK.E;
			2452.1979	K.KYGQDATNVGDEGGFAPNIQENK.E
ATP SYNTHASE B-	6.18	59713	1173.6724; 262.6395;	K.VVDLLAPYQR.G; R.TIAMDGTEGLVR.G;
SUBUNIT			1393.8010;	K.VLNTGAPITVPVGR.A;
(SPOT 8)			1425.7446;	R.VGLTGLTVAEYFR.D;
			1699.9394	R.LVLEVSHHLGQNVVR.T;
			2061.0296	R.QISELGIYPAVDPLDSTSR.M;
			2186.1362	R.IPSAVGYQPTLASDLGALQER.I;
			2539.2323	R.FEDQEGLPPIMTSLEVQDHPTR.L
